# Waiting for Triage: Unmeasured Time in Patient Flow

**DOI:** 10.5811/westjem.2014.11.22824

**Published:** 2014-12-08

**Authors:** Christopher Houston, Leon D. Sanchez, Christopher Fischer, Kathryn Volz, Richard Wolfe

**Affiliations:** Beth Israel Deaconess Medical Center, Department of Emergency Medicine, Boston, Massachusetts

## Abstract

**Introduction:**

The Centers for Medicare and Medicaid Services (CMS) requires reporting of multiple time-sensitive metrics. Most facilities use triage time as the time of arrival. Little is known about how long patients wait prior to triage. As reimbursement to the hospital may be tied to these metrics, it is essential to accurately record the time of arrival. Our objective was to quantify the time spent waiting to be triaged for patients arriving to the emergency department (ED).

**Methods:**

We conducted this study in an urban, academic, tertiary care center with approximately 54,000 annual ED visits. All patients arriving to the ED from November 1, 2012, to October 1, 2013, were enrolled. If patients didn’t go directly to a bed or triage, an observer greeted patients as they entered the ED and recorded the time of arrival. The triage time was recorded as normal. We calculated the difference between the arrival time and triage time.

**Results:**

There were 50,576 patient visits during the study period. Of these, 7,795 (15.4%) patients did not go directly to a bed or triage. For patients who waited for triage, median time from arrival to triage was 11 minutes (IQR 5–19, range 1–105). When stratified by the number of new patients who arrived in the ED in the previous hour, the percentage of greeted patients who waited more than 10 minutes for triage was: 0–5 new patients − 12.4%; 6–10 new patients − 48.8%; 11–15 new patients − 64.4%; 16+ new patients − 68%.

**Conclusion:**

Patients often waited more than 10 minutes to be triaged. As the number of patients registered in the previous hour increased, the percentage of patients who waited more than 10 minutes for triage increased significantly. During times of peak volume, 8.5% of all patients arriving to the ED waited more than 10 minutes for triage. This wait is not accounted for in the normal reporting of ED throughput times and metrics.

## INTRODUCTION

The Centers for Medicare and Medicaid Services (CMS) requires that hospitals report time-based metrics to evaluate emergency department (ED) performance. These include time from arrival to the ED to evaluation by a healthcare provider, to discharge or admission, and to various therapeutic interventions. It is expected that, in the near future, some of these metrics will determine Medicare reimbursement rates. The way times are recorded will thus need to be standardized to ensure compliance, as well as to provide a valid comparison between hospitals.

Most facilities use the time of initial triage and registration as the time of arrival. Triage commonly includes obtaining a chief complaint, vital signs, a brief history, and at times a review of recent ED visits and hospitalizations. This detailed triage provides important information but it takes time to perform. If multiple patients arrive simultaneously, there may be a delay in registering patients and recording the time to triage because of queuing. This unrecorded wait time prior to triage may cause significant underestimation of time-based metrics.

The objective of this study was to quantify the time spent waiting to be triaged for all patients arriving to the ED. It is our hypothesis that during times of peak volume, patients may spend a significant amount of time waiting to be triaged, time that is not captured, thus affecting throughput metrics. As reimbursement to the hospital may be tied to these metrics, it is essential to accurately record the time of arrival.

## METHODS

We conducted this study in an urban, academic, tertiary care center with approximately 54,000 annual ED visits. A determination was made that this project does not meet the federal definition of human subject research. All patients arriving to the ED from November 1, 2012, to October 1, 2013, were enrolled in the study in one of several ways. Emergency medical services (EMS) radio calls go directly to a bed where a physician and nursing staff meet them. Other EMS traffic as well as walk-in patients arrive to the ED and typically go directly to triage. In both of these instances, the triage time is the same as the arrival time. If all of the triage stations are occupied with patients, an observer greeted patients and ambulances as they entered the ED. The observer recorded the time of arrival and chief complaint. This information was listed on the tracking dashboard as “pre-triage.” An observer is present 24 hours a day, seven days a week. The triage time was recorded as normal. We calculated the difference between the arrival time and triage time. The two months preceding data collection was used as a trial period so all staff could become accustomed to this recording process and patients would not be missed.

## RESULTS

**Figure 1:**
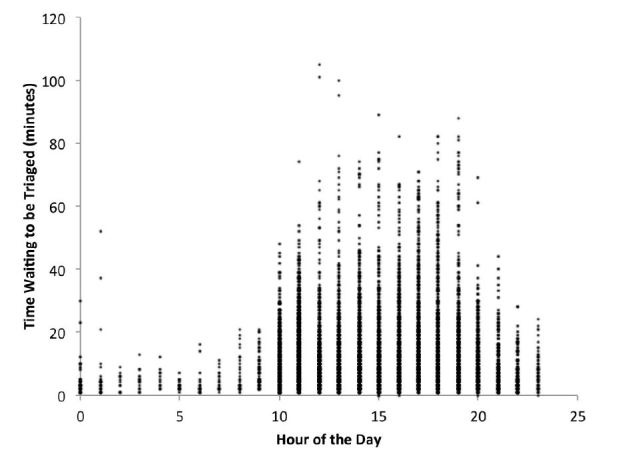
Time waiting for triage vs. time of day.

**Figure 2:**
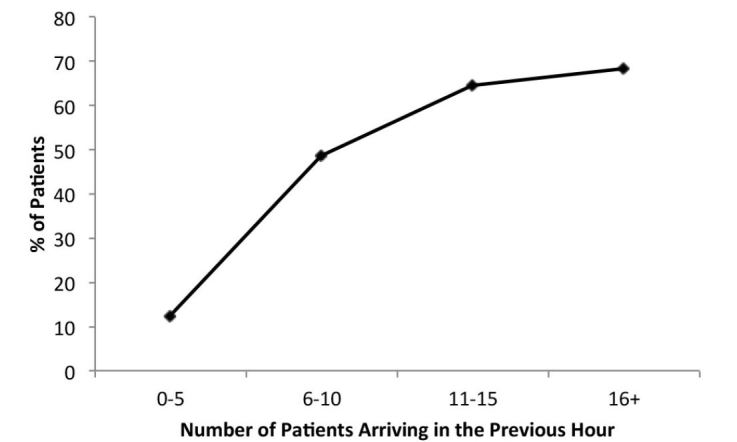
Percentage of patients waiting 10 or more minutes to be triaged with respect to the number of new patients arriving to the emergency department.

Of the 50,576 visits that occurred during the study period, 7,795 patients, 15.4% of all ED visits, waited to be triaged. There were patients who had to wait to be triaged at all hours of the day, but the longest wait times occurred between the hours of 10:00 and 20:00, which is when most EDs have the highest volume (Figure 1). For patients who waited to be triaged, wait times ranged from 1 to 105 minutes. The median time from arrival to triage was 11 minutes (IQR 5–19, range 0–105). 4,286 (8.5%) patients arriving to the ED waited 10 or more minutes to be triaged. Of those who waited for triage, 55% waited 10 or more minutes. When stratified by the number of new patients who arrived in the ED in the previous hour, the percentage of greeted patients who waited more than 10 minutes for triage was: 0–5 new patients − 12.4%; 6–10 new patients − 48.8%; 11–15 new patients − 64.6%; 16+ new patients − 68% (Figure 2).

## DISCUSSION

Little is known about how long patients wait prior to triage. There are multiple studies looking into the triage system, how it affects throughput, and ways to increase efficiency, but no studies specifically address this issue.^1^–^3^ A study by Weber et al.^4^ did record actual arrival times to the ED in order to look at whether or not mandatory triage identifies high-acuity patients within recommended time frames. They did not report times for patients of all acuity.

The door-to-doctor time is an important quality metric that has received increased scrutiny. With increased volume in EDs across the country, the goal of decreasing wait times has been difficult to accomplish. According to a 2009 paper by Horwitz,^5^ the time patients wait to see a doctor steadily increased from 1997 to 2006.

According to our data, patients often waited more than 10 minutes from the time of arrival to the ED until they were triaged. As the number of patients registered in the previous hour increased, the percentage of patients who waited more than 10 minutes for triage increased significantly as expected based on queuing theory. 8.5% of all patients arriving to the ED waited more than 10 minutes for triage. This wait is not accounted for in the normal reporting of ED throughput metrics, and may have an effect on quality of care. Our data implies that door-to-doctor times are longer than the standard method of reporting would indicate.

The Agency for Healthcare Research and Quality (AHRQ) has several pending quality measures that will be significantly affected by unaccounted time waiting for triage. The door to time of diagnostic evaluation by a qualified medical professional (door-to-doctor time) is a particularly important measure to patients as shown in previous studies.^6^,^7^ Numerous EDs already advertise real-time wait times on their websites. The methodology for the times advertised is normally not explained. Currently, there is no goal door-to-doctor time, but as more data is collected, there will surely be in the near future. The AHRQ mandates that these times be reported and published on the Medicare.gov website. As of November 2013, the mean national wait time is 28 minutes. The mean for Massachusetts is 38 minutes. The range is 8–35 minutes for the four tertiary care academic centers in the Boston area. When including community sites in the Boston area, the range is 8–50 minutes.^8^ One solution to the problem of having extended door-to-doctor times is to have a physician in triage. This has been studied at multiple sites but may not be feasible at all institutions.^9^,^10^

## LIMITATIONS

The major limitation of this study is that it was conducted at a single institution. Differences in the triage protocol, and the ratio of triage staff to patient volume will affect queuing times and may vary considerably from one institution to another. This study looked only at the time spent waiting for triage. Another question, which was not in the scope of this pilot study, is to determine if the patients who wait for triage have an increase in adverse events or bad outcomes. This will be addressed in future studies.

## CONCLUSION

In our study, we found that a significant number of patients are waiting 10 or more minutes for triage, which is approximately 30% of the mean national door-to-doctor time. We suspect that this phenomenon is not limited to our ED and suggest that this be studied in other locations. In our ED, we have begun tracking all patients as soon as they arrive to the ED and are listed as “pre-triage.” Recording arrival time accurately will be essential in ensuring that time-based metrics can be used to compare between institutions and that Medicare billing is compliant.

## References

[1] Arkun A, Briggs WM, Patel S (2010). Emergency department crowding: factors influencing flow. West J Emerg Med.

[2] Handel DA, Ma OJ, Workman J (2011). Impact of an expeditor on emergency department patient throughput. West J Emerg Med.

[3] Chan TC, Killeen JP, Kelly D (2005). Impact of rapid entry and accelerated care at triage on reducing emergency department patient wait times, lengths of stay, and rate of left without being seen. Ann Emerg Med.

[4] Weber EJ, Mcalpine I, Grimes B (2011). Mandatory triage does not identify high-acuity patients within recommended time frames. Ann Emerg Med.

[5] Horwitz LI, Bradley EH (2009). Percentage of US emergency department patients seen within the recommended triage time: 1997 to 2006. Arch Intern Med.

[6] Measuring Emergency Department Performance AHRQ Web site.

[7] Thompson DA, Yarnold PR, Williams DR (1996). Effects of actual waiting time, perceived waiting time, information delivery, and expressive quality on patient satisfaction in the emergency department. Ann Emerg Med.

[8] Hospital Compare Medicare website.

[9] Welch S, Davidson S (2010). Exploring new intake models for the emergency department. Am J Med Qual.

[10] Wiler JL, Gentle C, Halfpenny JM (2010). Optimizing emergency department front-end operations. Ann Emerg Med.

